# Along the Process Chain to Probiotic Tablets: Evaluation of Mechanical Impacts on Microbial Viability

**DOI:** 10.3390/pharmaceutics12010066

**Published:** 2020-01-15

**Authors:** Karl Vorländer, Ingo Kampen, Jan Henrik Finke, Arno Kwade

**Affiliations:** 1 Institute for Particle Technology, Technische Universität Braunschweig, Volkmaroder Straße 5, 38104 Braunschweig, Germany; i.kampen@tu-braunschweig.de (I.K.); jfinke@tu-braunschweig.de (J.H.F.); a.kwade@tu-braunschweig.de (A.K.); 2 Center of Pharmaceutical Engineering (PVZ), Technische Universität Braunschweig, Franz-Liszt-Straße 35A, 38106 Braunschweig, Germany

**Keywords:** compaction, formulation, freeze-drying, probiotics, *Saccharomyces cerevisiae*

## Abstract

Today, probiotics are predominantly used in liquid or semi-solid functionalized foods, showing a rapid loss of cell viability. Due to the increasing spread of antibiotic resistance, probiotics are promising in pharmaceutical development because of their antimicrobial effects. This increases the formulation requirements, e.g., the need for an enhanced shelf life that is achieved by drying, mainly by lyophilization. For oral administration, the process chain for production of tablets containing microorganisms is of high interest and, thus, was investigated in this study. Lyophilization as an initial process step showed low cell survival of only 12.8%. However, the addition of cryoprotectants enabled survival rates up to 42.9%. Subsequently, the dried cells were gently milled. This powder was tableted directly or after mixing with excipients microcrystalline cellulose, dicalcium phosphate or lactose. Survival rates during tableting varied between 1.4% and 24.1%, depending on the formulation and the applied compaction stress. More detailed analysis of the tablet properties showed advantages of excipients in respect of cell survival and tablet mechanical strength. Maximum overall survival rate along the complete manufacturing process was >5%, enabling doses of ${6\;\times \;10}^{8}$ colony forming units per gram (${\mathrm{CFU}\;g}_{\mathrm{total}}^{-1}$), including cryoprotectants and excipients.

## 1. Introduction

Production of dosage forms containing viable microorganisms is essential for the effective administration of probiotics. Probiotics are “live microorganisms, which when administered in adequate amounts confer a health benefit on the host” [1] and are well known from various dairy products and other functional food. Different diseases (e.g., inflammatory bowel syndrome, acute otitis media, irritable bowel syndrome, chronic lung diseases) were reported as potential targets for probiotics [2]. These pathologies are related to an imbalance of the human microbiome, which could be restored or prevented with administration of probiotics [2]. The key mechanisms of probiotic actions are very diverse. For example, they compete with pathogens for nutrients or binding sites, are involved in catabolism and production of vitamins and stimulate immune reaction or modulate inflammation [3]. Furthermore, some probiotic strains are able to produce antimicrobial bacteriocins [4]. Because of the displacement of pathogens and the secretion of antimicrobial substances, probiotics are considered as a promising alternative to antibiotics. Therefore, in the context of the increasing spread of antibiotic resistance, the pharmaceutical industry is getting more interested in such microorganisms [2]. The identification of suitable probiotics is one of the major tasks. Equally important and challenging is the production of suitable dosage forms containing viable microorganisms. Problems of liquid formulations are the acceptance by patients due to storage and application hurdles (e.g., cooled transport and storage, negative organoleptic properties, dosing) and the maintenance of the physiologically active state of the microorganisms. That is why even at low temperatures only short storage stability is observed [5]. To ensure long-term shelf-life, preservation is necessary. In the frozen state, microorganisms are physiologically inactive, and viability observed after freezing is preserved for a long time. Certainly, storage and transportation at subzero temperature is expensive. Another possible preservation method enabling long-term shelf-life and less costly storage is drying [2]. Until reconstitution, dried cells are physiologically inactive preserving their viability due to the low water activity [6]. Different drying techniques like vacuum drying, fluidized bed drying, spray drying and freeze-drying can be applied, with the latter being the most commonly used for drying sensitive biological materials like probiotics [2].

However, freeze-drying is a complex and expensive drying technique with detrimental effects on cells, especially because of ice crystal formation during freezing, so that protection strategies are required [6]. Hence, cryoprotectants reducing cell damage via vitrification, water replacement and preferential exclusion are added [2]. There are a lot of described cryoprotectants from various categories of substances, e.g., alcohols and alcohol derivatives, sugars and sugar alcohols, polymers, sulfoxides, amides and amines [7]. Even if, for example, trehalose usually provides a good protection, the most suitable cryoprotectant or combination of several is strain-specific and unfortunately no universal choice is known so far. That is the reason why often a high experimental effort is necessary to identify the best one or even a good one from all known cryoprotectants, or to identify novel ones.

Available probiotic products are divided into conventional, pharmaceutical formulations and non-conventional, i.e., food-based products, containing probiotics either due to their relevance in the production process or are added specifically to promote health benefits [6]. Among others, these include dairy products (e.g., milk, cheese, yogurts), chocolates as well as meat, and represent the majority of probiotic formulations. Indeed, these products were barely regulated for a long time. Viability at time of administration or when reaching the target site, mainly the intestinal tract, is questionable. However, for pharmaceutical application the regulation is much stricter [2] and, thus, more efforts are necessary for formulation development and characterization regarding the choice of best suited dosage form, the survival during processing and the storage stability to ensure the effectiveness of the administered probiotics.

For most probiotics, the target sites are the small intestine and the colon, and thus the oral delivery route is the most common and most effective [6]. Tablets are considered to be a well-suited solid dosage form for delivery of probiotics for different reasons. Firstly, being a dry dosage form, the probiotic microorganism’s viability in tablets is preserved and storage until administration is possible, at best for months or years at room temperature. Secondly, the properties of tablets can be controlled by usage of various excipients influencing the mechanical tablet strength as well as survival during compaction, storage and delivery to the target site [6,8]. Finally, tablets are the most common dosage form for oral administration, characterized by their high acceptance by patients as well as ease of administration compared to powders or liquids [6]. Indeed, problems can occur during tablet production, where microorganisms are harmed during drying, milling of dried cell aggregates and compression. The challenges of drying were mentioned before. Although the need of milling dried cell aggregates is mentioned by some workers as a problem because of the possibility of viability reduction, detailed knowledge regarding the influence on viability is still missing. In most cases, the cell damage due to the grinding step is often not quantified and even describing the procedure is frequently neglected or incomplete. During tableting, stresses due to compression, shear and heat occur, showing detrimental effects on microbial survival. The fact that large cells are damaged more strongly suggests mechanical stress (shearing) as the significant factor [9]. Besides dependency on cell size, the degree of damage also seems to be dependent on the compaction stress [10,11], compression speed [11], spatial distribution [12] and mechanical as well as physical properties of the used excipients, e.g., deformation characteristics [9,11,12,13] and particle size [10]. In general, increasing the compaction stress lowers the viability of tableted microorganisms independently of other influencing factors. However, the previously listed influencing factors determine the degree of damage related to the applied compaction stress.

**Figure 1:**
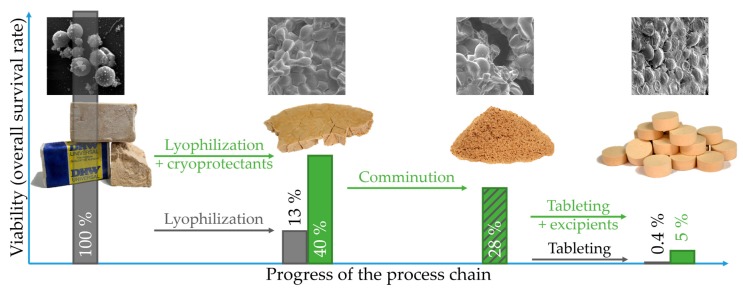
Process chain overview and process step specific influence on survival of *Saccharomyces cerevisiae* cells. Green color highlights increase of survival rate by addition of cryoprotectants and excipients. Cryoprotectants-containing lyophilizate was comminuted. Percentage values describe mean values.

In the present study, the baker’s yeast *Saccharomyces cerevisiae* was used as the model organism. Firstly, the cells were freeze-dried with various cryoprotectants and freezing temperatures. Secondly, the lyophilizates were comminuted and partly mixed with direct compaction excipients. Thirdly, formulations were tableted at different compaction stresses and tablet properties were determined. An overview is provided in Figure 1. The objective of this study was the quantification of survival rates for every single process step in order to identify the critical steps during production as well as to evaluate the influence of selected formulation and process parameters.

## 2. Materials and Methods 

### 2.1. Chemicals

Baker’s yeast *S. cerevisiae* (Deutsche Hefewerke GmbH, Nürnberg, Germany) was used. A sufficient amount of the same batch for all experiments was stored at −20 °C until use. For all experiments, cells were suspended in purified water, 1/5 × PBS (isotonic phosphate-buffered saline solution, pH 7.4) (Sigma-Aldrich Chemie GmbH, München, Germany) or purified water containing cryoprotectants, respectively. Cryoprotectants dextran 20, glycerin and skimmed milk powder were purchased from Carl Roth GmbH + Co. KG (Karlsruhe, Germany), l-Glutamic acid and α-Lactose monohydrate from Sigma-Aldrich Chemie GmbH (München, Germany), d(+)-Maltose monohydrate from AppliChem GmbH (Darmstadt, Germany) and trehalose dihydrate from intelligent sugar GmbH (Otzberg, Germany).

For determining colony forming units, yeast extract peptone dextrose (YPD) agar plates with 20 ${g\;L}^{-1}$ of peptone ex casein, 10 ${g\;L}^{-1}$ of yeast extract, 22 ${g\;L}^{-1}$ of glucose monohydrate and 15 ${g\;L}^{-1}$ of Agar-Agar Kobe 1 were used, all chemicals supplied by Carl Roth GmbH + Co. KG (Karlsruhe, Germany).

Direct compression excipients used were microcrystalline cellulose (MCC) (Vivapur 102, JRS Pharma GmbH + Co. KG, Rosenberg, Germany), dicalcium phosphate (DCP) (Emcompress Anhydrous, JRS Pharma GmbH + Co. KG, Rosenberg, Germany) and lactose (Tablettose 70, Meggle-Pharma, Wasserburg am Inn, Germany).

### 2.2. Viability Assay

For determining viability, the method of counting colony forming units (CFU) was applied by default. Depending on the evaluated process step, thawed yeast cells were suspended in PBS. Lyophilizates, blends and tablets were reconstituted in PBS at room temperature for at least 30 min on a see-saw. Cell suspensions were serially diluted until reaching a suitable concentration and spread onto yeast extract peptone dextrose agar plates. Always triplicates were spread out and after incubation for 48 hours at 28 °C, colonies were counted manually. Colony forming units were calculated and related to cell dry weight (CDW) or total weight (e.g., tablet weight including the mass of the cells as well as excipients and cryoprotectants).

Survival rates were used to characterize the loss of viability for single process steps and were calculated according to the following equation:

(1) $$\mathrm{Survival}\;\mathrm{rate}\;\left[\%\right]=\frac{\mathrm{CFU}\;\mathrm{per}\;\mathrm{CDW}\;\mathrm{after}\;\mathrm{process}\;\mathrm{step}}{\mathrm{CFU}\;\mathrm{per}\;\mathrm{CDW}\;\mathrm{before}\;\mathrm{process}\;\mathrm{step}}\;\times \;100.$$

### 2.3. Freeze-Drying

To investigate the influence of freezing temperature on survival during freeze-drying, thawed *S. cerevisiae* cells were suspended 1:1 (*w*/*w*) in purified water, a solution of 50 ${g\;L}^{-1}$ of skimmed milk powder and 50 ${g\;L}^{-1}$ of skimmed milk powder together with 100 ${g\;L}^{-1}$ of trehalose, respectively. Suspensions were frozen at three different freezing temperatures (−20 °C and −35 °C in the freezer and −196 °C in liquid nitrogen), stored at −20 °C, −35 °C or −80 °C for 24 h, respectively, and subsequently freeze-dried (Beta 2–8 LSCplus, Martin Christ Gefriertrocknungsanlagen GmbH, Osterode am Harz, Germany). Primary drying took 24 h and was done with a continuous temperature increase (1.25 °C per hour until shelf temperature was 0 °C, then 2.5 °C per hour until final shelf temperature of 20 °C was reached) at 0.220 mbar. For secondary drying, pressure was set to 0.002 mbar at 20 °C for 24 h.

In addition, to investigate the influence of different cryoprotectants on survival during freeze-drying, thawed cells were suspended 1:1 (*w*/*w*) in solutions of cryoprotectants (dextran 20, glycerin, skimmed milk powder, l-Glutamic acid, α-Lactose monohydrate, d(+)-Maltose or trehalose dihydrate) with three different concentrations related to final concentrations of 0.40, 0.25 and 0.15 ${g\;g}_{\mathrm{CDW}}^{-1}$ and incubated for 1 h at 28 °C in shaking flasks. Besides, combinations of 0.25 g of trehalose together with 0.25 g and 0.15 g of skimmed milk powder per gram CDW, respectively, were used as well as 0.15 g of trehalose plus 0.15 g of skimmed milk powder per gram CDW. Purified water as well as 1/5 × PBS solution were used as reference suspending media. Suspensions were frozen at −20 °C and subsequently freeze-dried as described above. For all tested conditions, colony forming units were determined.

### 2.4. Milling of Lyophilizates

The lyophilizates were comminuted using a vibratory sieve shaker (AS 200 control, Retsch GmbH, Haan, Germany). Lyophilizates were placed on a sieve with a 500 µm mesh size and fragmented by vibration, varying the amplitude between 0.5 and 3.0 mm. For the different used amplitudes, colony forming units were determined.

### 2.5. Blending with Excipients

Lyophilized yeast cells were blended with MCC, DCP or lactose, respectively. After adding 20 wt% of the excipient to the comminuted lyophilizates, mixing was carried out with a 3D shaker mixer (TURBULA, Willi A. Bachofen AG, Muttenz, Switzerland) for 2 min at 49 $\min^{-1}$. The batch size of each blend was 100 g and the fill level of the mixing vessels approx. 50%.

### 2.6. Tablet Preparation

Yeast cells lyophilized with 0.15 g of skimmed milk powder and 0.25 g of trehalose per gram CDW (below shortened as FDY_MT_) were used for tablet production. Freeze-dried cells were either compacted without excipients or after addition of MCC, DCP and lactose, respectively. Tablets with a mass of 500 mg were produced displacement controlled using a compaction simulator (Styl’One evolution, Medelpharm, Beynost, France), Analis acquisition software (Medelpharm, Beynost, France) and a flat punch/die pair with a diameter of 11.28 mm (EURO-D, Adamus S.A., Warszawa, Poland) were used. Punch distance was varied to obtain compaction stresses of 100, 150, 200, 300 and 400 MPa, respectively. Die-filling was achieved using a paddle forced feeder (300 rpm). A generic compression profile with a linear punch movement (Filling: 3.0 s, Compression: 0.2 s, Decompression: 0.3 s) and, depending on compaction stress, with correlating real compression times between 105 ms and 130 ms and dwell times between 34 ms and 42 ms was used.

### 2.7. Tablet Characterization

Tablets were characterized according to the European Pharmacopoeia [14]. 24 h after production, tablet mass was determined by weighing, and geometric dimensions and breaking force were acquired using a manual tablet tester (MultiTest 50, Sotax AG, Aesch, Switzerland) (Ph. Eur. 9.3 2.9.8). Moreover, a disintegration test was performed (DisiTest 50, Sotax AG, Aesch, Switzerland) (Ph. Eur. 9.3 2.9.1) and friability was tested (PTF, Pharma Test Apparatebau AG, Hainburg, Germany) (Ph. Eur. 9.3 2.9.7). In addition, viability of the compacted yeast cells was analyzed following the previous description. Further, geometric parameters were used to calculate tablet tensile strength $\sigma_{t}$ as

(2) $$\sigma_{t}\;=\;\frac{2\;F}{\pi \;d\;h},$$

where F is the breaking force, d is the tablet diameter and h is the tablet thickness [15]. For calculation of tablet porosity ε, the true density $\rho_{\mathrm{true}}$ of the formulations was determined by helium gas pycnometry (Ultrapyc 1200e, Quantachrome Instruments, Boynton Beach, FL, USA). Together with the tablet geometric properties, tablet porosity was determined mathematically as

(3) $$\varepsilon \;=\;\frac{\rho_{\mathrm{true}}}{\frac{\pi }{4}\;d^{2}\;h}.$$

### 2.8. Scanning Electron Microscopy

Scanning electron microscopy (SEM) images were taken with a Helios G4 CX (FEI, Hilsboro, OR, United States). Lyophilized cells (without cryoprotectants) and compressed lyophilized cells (without cryoprotectants and excipients) were investigated. All samples were sputtered with platinum (LEICA EM ACE600, Leica Microsystems GmbH, Wetzlar, Germany).

## 3. Results and Discussion

The effects of freeze-drying (e.g., cryoprotectants), milling lyophilizates, mixing of *S. cerevisiae* with excipients as well as tableting (e.g., compaction stress, excipients) on microbial viability and tablet properties were investigated. The results were used to specify critical process steps and parameters to identify the first viability improvement approaches.

### 3.1. Influence of Freeze-Drying on Viability

#### 3.1.1. Freezing Temperature

**Figure 2:**
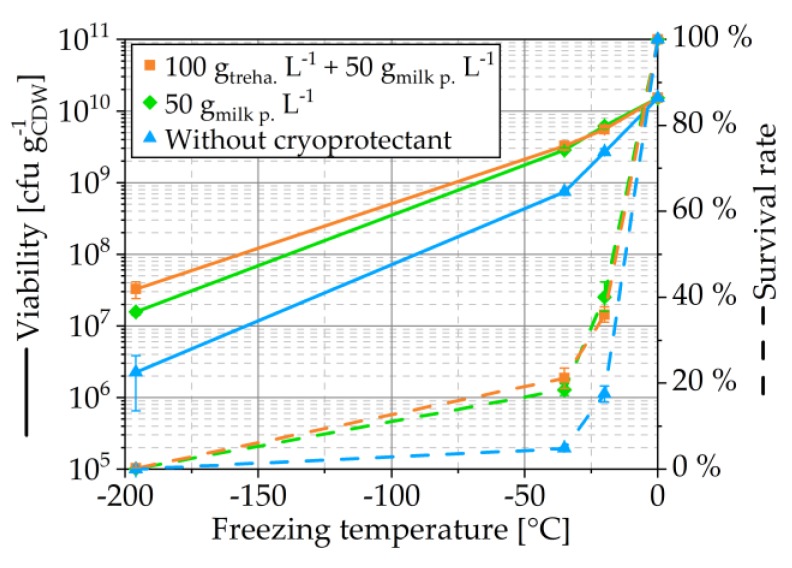
Dependency of viability and survival rate on freezing temperature. Cell concentration was 150 $g_{\mathrm{CDW}}{\;L}^{-1}$. Symbols represent means and error bars standard deviation; *n* = 3.

As seen in Figure 2, the freezing temperature has a drastic influence on yeast cell survival after lyophilization. Independent of the addition of cryoprotectants, the viabilities and survival rates were lower with a lower freezing temperature for the tested temperatures. When cell suspensions were frozen in the freezer, freezing took approximately 20 min and 50 min at −35 °C and −20 °C, respectively, and freezing in liquid nitrogen was completed in less than a minute. Therefore, different freezing rates can be concluded, each of which has a specific effect on the survival of the yeast cells.

In general, too low freezing rates are associated with cellular damage due to osmotic stress [16]. The formation of extracellular ice crystals causes a concentration of solved compounds. The consequence is a dehydration of the cells until the intra- and extracellular concentrations are equalized. The incessant growth of extracellular ice increases the concentration further and increases dehydration of cells. The continual dehydration of the cells and the related concentration causes osmotic stress that could reach a lethal extent [16]. When freezing occurs fast, but too slow for vitrification, the formation of intracellular ice crystals is more likely and causes lethal mechanical destruction. The optimal freezing temperature is in-between both borders. Theoretically, vitrification of the cells could be an alternative, but regarding the survival rates, it can be seen in Figure 2 that freezing in liquid nitrogen was not fast enough for vitrification. This is only possible for very small volumes and even with high-pressure freezing, vitrification of only a few microliters is possible. However, freezing and lyophilization of such small amounts is not relevant for the production of probiotics and would not even have yielded enough material for subsequent experiments in the present study. Even if vitrification was not reached, the freezing rate was still very high, and thus the formation of intracellular ice crystals is plausible and drastically reduced the viability. With both tested lower freezing temperatures and rates, higher viabilities and survival rates were observed. The lowest cell damage occurred with the lowest freezing rate.

However, it is important to mention that the different final temperatures affected the viability, too. Additional experiments showed that the viability was reduced when cells frozen at −20 °C were subsequently cooled with liquid nitrogen (LN_2_). The survival rates (determined after thawing) during the stepwise temperature decrease were between both single-step freezings. When the survival rates were determined, not after freezing and thawing but after freezing as described followed by lyophilization, the same trend was observed with a much higher loss of viability when LN_2_ was used, especially when the freezing was done directly in LN_2_ and not stepwise. For all further experiments, freezing was done at −20 °C. Abadias et al. also identified this temperature as the best freezing temperature for lyophilization of the yeast *Candida sake* (freezing at −12 °C, −20 °C, stepwise at −12 °C and −20 °C as well as in liquid nitrogen was compared) [17]. Therefore, probably, a higher freezing temperature for the lyophilization of *S. cerevisiae* would not show a significant positive effect on cell survival.

#### 3.1.2. Cryoprotectants

**Figure 3:**
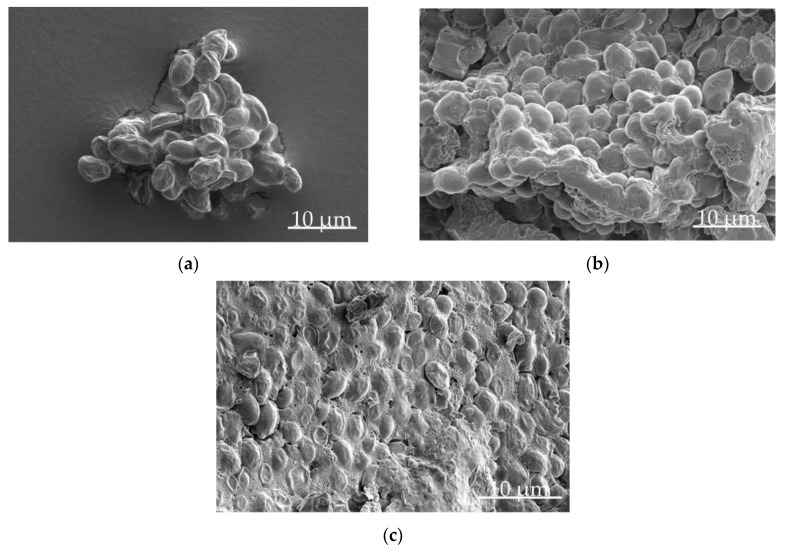
Scanning electron microscopy images of lyophilized yeast cells suspended in purified water (**a**) or a solution of trehalose and milk powder (**b**). The third image (**c**) shows the fracture surface of a tablet composed of compacted cells freeze-dried with trehalose and milk powder. Compaction stress was 200 MPa.

Table 1 shows the absolute viability and relative survival rates of yeast cells which were freeze-dried with different cryoprotectants with varied concentrations. Only 12.4% of cells suspended in purified water (SEM image is shown in Figure 3a) and 13.5% of cells suspended in 1/5 × PBS solution survived the lyophilization procedure, showing the need for adding cryoprotectants. This problem of low survival rates after lyophilization is well known for the majority of microorganisms and a lot of research was and is done to identify suitable cryoprotectants. Unfortunately, no ideal global additive is known so far. However, for freeze-drying of *S. cerevisiae*, survival rates for lyophilization with different cryoprotectants and freezing rates were published [18,19,20,21].

In the present study, the highest viability was found for addition of 0.4 g of milk powder per gram CDW with $\left(7.50\;\pm \;1.73\right){\;\times \;10}^{9}{\;\mathrm{CFU}\;g}_{\mathrm{CDW}}^{-1}$ (mean ± SD), which corresponds to a survival rate of 42.9% (SEM image is shown in Figure 3b). Obviously, that is less than the 96% reported by Berny and Hennebert [21] that, among others, can be attributed to strain-specific tolerance and different experimental conditions. Due to the lower standard deviation in the case of trehalose addition and the accompanying lower variance in all subsequent experiments, the combined addition of milk powder and trehalose was considered as more suitable for analysis of further process steps, still reaching survival rates of approximately 40%. Since for the final product the higher viability related to the total mass after lyophilization is essential, 0.25 g of trehalose and 0.15 g of milk powder per gram CDW were used as cryoprotective additives for analysis of subsequent process steps. Indeed, with 0.15 g of dextran per gram CDW, the viability related to lyophilizate mass was higher, but the mean variation was again very high and would affect the evaluation of subsequent experiments.

The observed differences in suitability of the tested agents to protect biological structures like the viability of cells during freezing and drying depends on their specific protection mechanisms. First of all, depending on their physico-chemical properties, protectants are differently permeable to the cells. On the one hand, this determines the protective mechanism, but also the efficiency. Especially compounds stabilizing intra-cellular structures must be absorbed by the cells before the freezing process begins. For example, skim milk is not absorbed by microorganisms but forms a protective layer on the cell surface whereas other protectants may permeate the cell wall or cell membrane [22]. This applies to sugars such as trehalose, maltose or lactose, which in general enhance the glass transition temperature, thereby restricting the molecular mobility and thus stabilizing the cells during freeze-drying [23,24].

During freeze-drying, structural changes of membrane lipids and sensitive proteins occur because of the removal of hydrogen-bonded water from the phospholipid headgroups. This brings the alkyl chains close together, causing increased van der Waals interactions and thus lamellar phase changes to the gel phase [25]. Upon rehydration, cell integrity is lost due to packaging defects [25]. Trehalose is a natural cryoprotectant that directly interacts with the polar headgroups of the lipids. During drying, these interactions replace those of water molecules (water replacement hypothesis), minimizing the probability of formation of the critical gel phase and loss of cell integrity after rehydration [25]. In addition, trehalose has a high glass transition temperature, and thus reduces the denaturation of embedded proteins due to reduced molecular mobility (unfolding and aggregation) [23,25]. Maltose also has a high glass transition temperature and is suspected to directly interact with the lipid headgroups. However, protection was less compared to the chemical isomer trehalose. An explanation for this is the higher flexibility between both monomers of trehalose, enabling a better interaction with the phospholipid headgroups [25]. 

In most cases, a higher concentration of protective substances was better suited to maintain cell viability during lyophilization. However, when using dextran or glycerin an opposite effect of the protectant concentration can be observed. Both of them significantly increase the viscosity of the extracellular medium. During extracellular ice formation, besides dehydration of the cells, the viscosity further increases. This reduces the rate at which intracellular water could be drawn back from the cell to ice crystals [26]. A too high viscosity is suspected to hinder the partial dehydration of the cells. To a certain extent, however, this would be beneficial to prevent a lethal increase in volume during freezing.

### 3.2. Influence of Lyophilizate Comminution on Survival

To achieve a flowable powder, lyophilizates were comminuted by vibration on a sieve with a mesh size of 500 µm. During vibration, the lyophilizates were abrasively stressed and fragmented into smaller particles. The amplitude was varied in a range of 0.5 mm to 3.0 mm but no significant influence (ANOVA, α = 0.05) of amplitude on viability after milling and an average survival rate of 71 ± 4% was observed. Probably, the decrease in viability is the result of cracks, which do not only pass through the cryoprotectant matrix, but also run through and break the cells themselves. This could be a problem especially when the surrounding matrix is harder than the cells and the cells act like imperfections in this composite material, comparable to flaws and imperfections in general strength theory [27].

### 3.3. Influence of Mixing with Excipients on Survival

**Figure 4:**
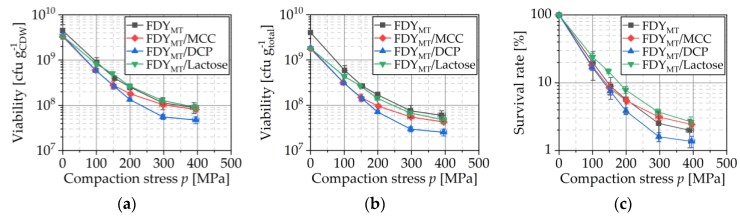
Effect of compaction stress on viability related to the cell dry weight (**a**) and total mass (**b**) as well as on the survival rate (**c**). Freeze-dried yeast cells with 15% milk powder and 25% trehalose as cryoprotectants (FDY_MT_) without further excipients and mixed with MCC, DCP and lactose were compacted. Symbols represent means and error bars standard deviation; *n* = 3.

During dry mixing with excipients, the lyophilized yeast cells were subjected to mechanical stress because the comminuted cell agglomerates rub against each other, excipient particles and walls. The viability of the FDY prior mixing was (4.5 ± 1.6)$\;\times \;10^{9}{\;\mathrm{CFU}\;g}_{\mathrm{CDW}}^{-1}$ and was reduced to (3.40 ± 0.09)$\;\times \;10^{9}{\;\mathrm{CFU}\;g}_{\mathrm{CDW}}^{-1}$ averaged for the three blends (viability data in Figure 4a for a compaction stress of 0 MPa). Deduced from this, the survival rate during this step was 76 ± 28%, independent of the used excipient, which would mean that this procedure would be equally detrimental as the milling step. However, this does not seem plausible due to gentle conditions and short duration of mixing compared to the abrasion that was exerted during the previous milling. Additional experiments showed that the decrease is not so high. Mixing the lyophilized yeast cells alone or together with DCP did not reduce the viability significantly even after mixing times up to 6 h. Considering the 15-min mixing time, the discrepancy in viability at 0 MPa is merely the consequence of an uncertain determination of the initial viability of the freeze-dried yeast before the addition of excipients ((4.5 ± 1.6)$\;\times \;10^{9}{\;\mathrm{CFU}\;g}_{\mathrm{CDW}}^{-1}$). A cause for this high standard deviation could not be identified and a verifying re-determination has not been possible due to the decrease in viability during storage. However, the standard deviation of 36% explains the high uncertainty of the survival rates calculated for the mixing, which are based on this mean and standard deviation (propagation of uncertainty).

### 3.4. Influence of Tableting Stress and Formulation on Survival and Physical Tablet Properties

#### 3.4.1. Viability Curves

Powdered lyophilizates (freeze-dried yeast with 15% milk powder and 25% trehalose as cryoprotectants, FDY_MT_) were either compacted with or without (pure FDY_MT_) previous mixing with microcrystalline cellulose (FDY_MT_/MCC), dicalcium phosphate (FDY_MT_/DCP) and lactose (FDY_MT_/Lactose), respectively. Figure 3c shows an SEM image of an FDY_MT_ tablet. Although no widespread fragmentation is visible, a deformation of the tableted cells compared to the cells before compaction is obvious. During this deformation, cellular structures were harmed and not all cells survived this stress resulting from compaction. The profiles of viability depend strongly on the applied compaction stress as shown in Figure 4. With increasing compaction stress, the viability decreased for all tested formulations. For example, survival rates dropped from 19.7% to 2.0% for pure FDY_MT_ tablets, while compaction stress raised from 100 to 400 MPa (Figure 4c).

This is in line with expectations and the results published by other authors [10,28,29,30]. For instance, Plumpton et al. found a logarithm decrease of survival rates over three decades when *S. cerevisiae* was compressed with up to 280 MPa. The reason for this dependency is the increase of shear and compression stress as well as local heat with increasing compaction stress. However, how strong the viability was affected by the compaction stress was different because of its dependency of formulation aspects, here namely the added excipients. The lowest viability was observed when DCP was added (the corresponding survival rate was 1.4% for compaction stress of 400 MPa), followed by the addition of MCC (FDY_MT_/MCC), showing a 2.4% survival for compaction stress of 400 MPa. With regard to the CFU per gram CDW, the viabilities of FDY_MT_ and FDY_MT_/lactose tablets were very similar for the tested compaction stresses. Indeed, survival rate after tableting with 400 MPa compaction stress was a bit higher when lactose was used (2.6%). This indicates an additional damage during the tableting process due to the DCP particles that is probably the result of movement, deformation and fracture of these particles, causing extra stress on lyophilized cells. This is in accordance with the findings of highest mean yield pressures for DCP as compared with other excipients [31], displaying the stiffest material behavior and the highest tendency to deform by fragmentation, for DCP. The extent of this disadvantage is further enhanced by the 20% lower number of cells in the three formulations with excipients as demonstrated in Figure 4b. The total weight of the produced tablet and therefore the colony forming units related to this weight were calculated to compare the efficiency of the final product. However, independently of the tested excipients, the CFU per gram total weight was lower for every compaction stress, indicating that the usage of the excipients as a method for maximization of viability is questionable regarding the concentration of viable cells and derivable knowledge, especially when DCP is used.

#### 3.4.2. Compression Analysis

**Figure 5:**
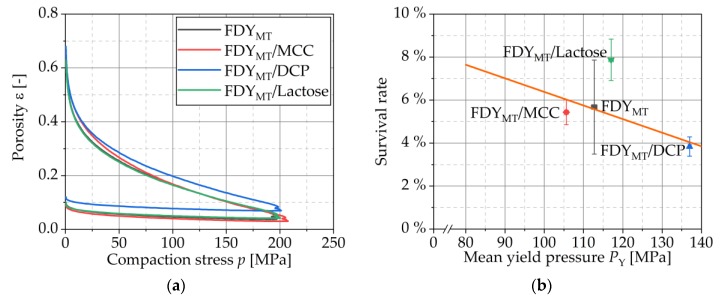
(**a**) In-die porosity profiles of pure FDY_MT_ and FDY_MT_ mixed with MCC, DCP and lactose. (**b**) Correlation of mean yield pressure of formulation and survival of cells during tableting with a compaction stress of 200 MPa. The R^2^ of the linear regression is 0.220. Symbols represent means and error bars standard deviation; *n* = 3.

The previously discussed properties and differences could be the consequence of different compaction behaviors of the added excipients. The Heckel equation is the most frequently used compaction equation [32,33] and describes the relationship between porosity of a tablet and the applied stress [34]:

(4) $$\ln\left(\frac{1}{\varepsilon }\right)=\;k\;\cdot \;p\;+\;A$$

where ε is the porosity, k is the Heckel constant and p is the applied stress. The second constant A describes the particle movement during initial compaction phase and is a measure of pre-compaction [35] and is dependent on particle properties, e.g., size, form and hardness [36]. The reciprocal value of the slope k is the mean yield pressure $P_{Y}$ [37]. It quantifies the tension that is necessary to induce plastic flow of the substance and thereby characterizes the intrinsic resistance of the material against the acting stress. It is further interpreted to characterize the deformation behavior. The basic assumption for the Heckel equation is that the densification of the powder can be described with a kinetic of the first order [34]. The determination of k and A is fulfilled by linear regression of porosity profiles plotted as ln(1/ε) as a function of the compaction stress p (non-logarithmized porosity plots are shown in Figure 5a). Mean yield pressures were 112.7 ± 0.7, 105.6 ± 0.5, 137.0 ± 0.4 and 117.0 ± 0.6 MPa for FDY_MT_, FDY_MT_/MCC, FDY_MT_/DCP and FDY_MT_/Lactose, respectively. Since data were randomly distributed (R^2^ of 0.220 for linear regression) in Figure 5b, no correlation was found between the mean yield pressure of the formulation and the survival rate. This is in line with observations that were reported for a strain of *Lactobacillus rhamnosus* where the bacterial cell survival was not correlated to the mean yield pressure of different filler–binders [13].

From mean yield pressures, deformation behavior can be interpreted. Materials with a high mean yield pressure are assumed to be hard/brittle whereas materials with a low mean yield pressure are assumed to be soft/plastic. The mean yield pressures of pure substances MCC, DCP and lactose were determined to be 92.9 ± 0.2, 524 ± 5 and 172.7 ± 0.3 MPa, respectively. Accordingly, MCC deforms plastically, DCP; on the other hand, brittle and lactose combines both deformation characteristics. For the considered formulations, however, the difference is less pronounced due to the low mass fraction of excipients and all mean yield pressures are in the magnitude of FDY_MT_, but it yet displays the same order as for the pure excipients. It is probable that higher mass fractions of the excipients would show an influence of the excipients’ deformation behavior and therewith on the survival rates. This opens a new area of understanding that must be evaluated in systematic studies in the future.

#### 3.4.3. Elastic Recovery

**Figure 6:**
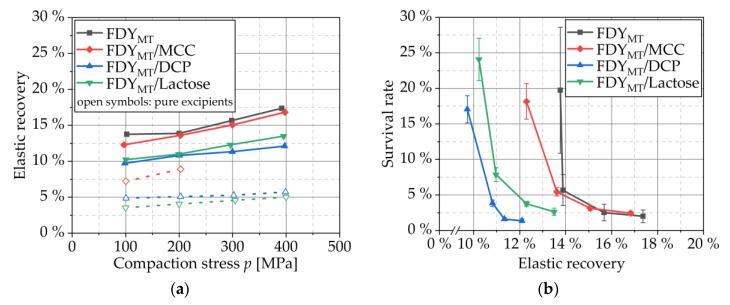
(**a**) Elastic recovery profiles of FDY_MT_, FDY_MT_ mixed with MCC, DCP and lactose as well as of the pure excipients. Symbols represent means; *n* = 10. (**b**) Dependency of survival rate on elastic recovery. Symbols represent means and error bars standard deviation; *n* = 3.

At the highest compaction stress, powders reach a minimum height with minimum porosity. During relaxation, elastic re-deformation occurs depending on the material and process. For this, the elastic recovery is a measure that relates the difference between out-die tablet height and minimum in-die tablet height to the minimum in-die tablet height. The compaction stress-dependent profiles of the elastic recovery of FDY_MT_, FDY_MT_ mixed with the excipients MCC, DCP and lactose as well as of the pure excipients is shown in Figure 6a. FDY_MT_ shows a significantly higher elastic recovery than the pure excipients. The addition of MCC hardly affects the elastic recovery, whereas it is reduced by the addition of DCP or lactose. The relationship between the degree of elastic recovery and survival rate during direct compression of *L. rhamnosus* GG was described by Byl et al. They found higher survival rates when the degree of elastic recovery was higher: When less elastic DCP was used, survival rate was approx. 30% compared to approx. 70% when more elastic MCC was added [13]. However, considering Figure 6b, the global and sole dependency of survival rate on elastic recovery or such a dependency exceeding the influence of the formulation were not found in this study. On the contrary, when examining the data points for each formulation, low elastic recovery appears to be advantageous. This is due to a superposition with the influence of compaction stress (which itself causes higher elastic recovery when elevated) within each formulation. The shift of the elastic recovery-dependent survival rate curves shows that the survival is material-specific but cannot be expressed as a general and direct function of elastic recovery.

#### 3.4.4. Compressibility

**Figure 7:**
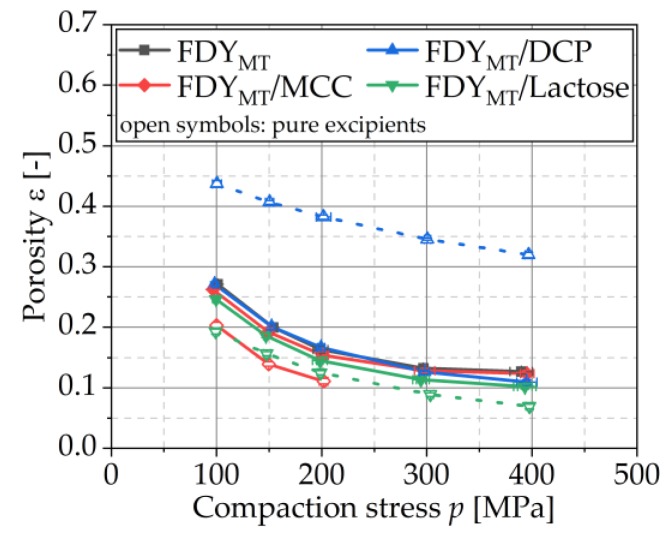
Compressibility profiles of FDY_MT_ and FDY_MT_ mixed with MCC, DCP and lactose as well as of pure excipients. Symbols represent means and error bars standard deviation; *n* = 10.

**Figure 8:**
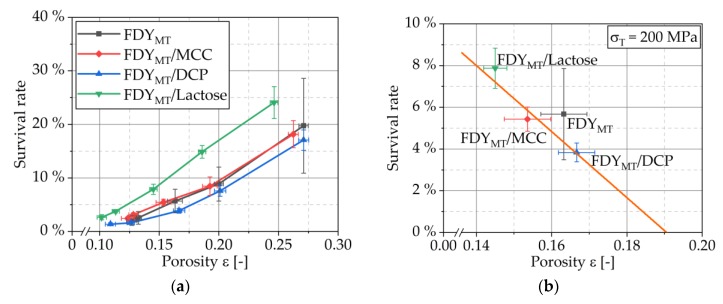
Survival rates depending on porosity for formulations of pure FDY_MT_ and FDY_MT_ mixed with MCC, DCP and lactose compressed with various stresses (**a**) and selectively magnified for 200 MPa (**b**), respectively. Symbols represent means and error bars standard deviation; *n* = 3.

**Figure 9:**
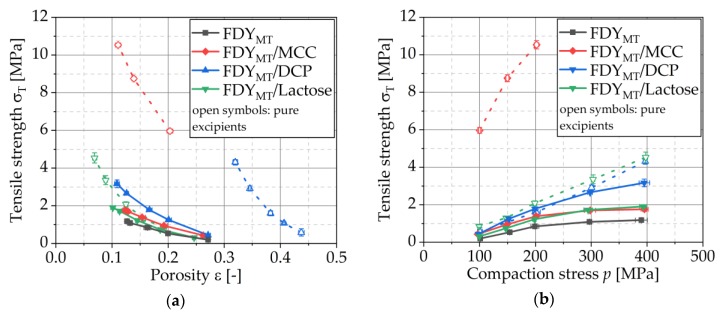
Compactibility (**a**) and tabletability profiles (**b**) of FDY_MT_ and FDY_MT_ mixed with MCC, DCP and lactose as well as of pure excipients. Symbols represent means and error bars standard deviation; *n* = 10.

Compressibility profiles (definition, e.g., [33]) are shown in Figure 7 for compaction of pure FDY_MT_ and MCC, DCP and lactose as well as of FDY_MT_ mixed with these excipients. For all tested blends, the porosity decrease with increased compaction stress is comparable. However, the porosity was slightly lower when MCC, DCP and lactose were added, respectively. Figure 8 relates the porosity with the cell survival. For all formulations, the survival rates decreased with decreasing porosity because of the increased compaction stress. However, the degree of this influence showed to be material specific, which explains the seemingly opposite observation of higher survival rates (R^2^ = 0.913 for linear regression) for lower porosities in Figure 8b. This must be correlated to the deformation behavior of the respective excipients as these are the only addition to the FDY_MT_. The stiffest excipient DCP yields porosities of approx. 30% at a maximum stress of 400 MPa without the addition of FDY_MT_, while pure MCC and lactose yield porosities smaller than 7% in this stress range [31]. Accordingly, the FDY_MT_ dominates the compressibility of the formulation, resulting in porosities of 10–13% at 400 MPa. This implies two possible mechanisms for the case of DCP: (i) DCP is forming a porous, coherent network that incorporates FDY_MT_ particles and (ii) DCP particles penetrate FDY_MT_ particles. On the one hand, the formation of the framework of DCP particles enables the production of the strongest tablets. On the other hand, with increasing compaction stress, the framework is further compressed, leading to fracture of the brittle DCP particles. Arising sharp-edged fragments penetrate or cut through the cells. The determination of cell survival (Figure 4) and tensile strength (Figure 9) can assist the evaluation of these hypotheses.

#### 3.4.5. Compactibility and Tabletability

Compactibility is defined as the relationship between the tensile strength and porosity of the tablets [33]. In Figure 9a, compactibility profiles are given for compaction of pure FDY_MT_, excipients and FDY_MT_ mixed with the excipients. All tested formulations showed an exponential decrease in tensile strength with increasing porosity, which is typical and the logical consequence of weakening compacts by larger or more pores [38]. 

The correlation of tensile strength and compaction stress finally shows the ability of a powder to form a tablet with a certain strength when a certain stress is applied and therefore is termed tabletability [39]. Tabletability profiles presented in Figure 9b show for all compaction stresses that the ability to form a tablet with high tensile strength was improved when MCC, lactose and especially DCP were added, respectively. For all investigated formulations, the tensile strength increased with higher compaction stress. This behavior was expected on the basis of the previous shown compressibility and compactibility data and was in accordance with the literature [40].

In general, depending on tablet formulation, tensile strength continues to increase with compaction stress, but gradually levels off at higher stresses or even decreases due to overcompression. For the studied formulations and stress ranges, no overcompression was observed but it seems that an approximation of a maximum tensile strength was almost reached, especially for pure FDY_MT_ and FDY_MT_/MCC, respectively. The pure excipients DCP and lactose, on the other hand, still show a linear trend of tensile strength in the considered compaction stress range. Even if the tensile strength of the yeast lyophilizate tablets could be increased by the addition of excipients, the tabletability seems to be largely determined by the lyophilizate particles, which is why all mixtures with FDY_MT_ show similar plots.

However, as a pure excipient, MCC displays a significantly higher tensile strength than DCP and lactose when compacted under the same stress (Figure 9b). To understand the observed tabletability profiles, the interaction of FDY_MT_ and excipients accordingly plays a crucial role. Ductile materials such as MCC develop high binding forces based on the formation of large contact areas between the particles without fragmentation. The specific surface area of these particles is only changed to a low extent and they remain in their relative position within the fabric. Such contacts formed by ductile deformation can be weakened by FDY_MT_ with lower bonding potential surrounding these particles, virtually shielding them from contacts to other MCC particles. This is comparable to the susceptibility of such materials to lubricants, which significantly reduce tensile strength by layering the excipients particles surface causing an impairment of the formation of strong excipient–excipient interactions [41]. In contrast to that, the extraordinary elevation of tensile strength by DCP fosters the hypothesis that a coherent network of DCP was formed. This is assumingly based on the brittle fracture and, by that, enabled displacement of new fragments within the matrix of FDY_MT_, finally allowing the formation of such a coherent excipient network. A second approach to explain the exceptional strength of FDY_MT_/DCP tablets is based on the formation of a particularly solid composite, which is enabled by combining the ductile yeast lyophilizate with the brittle DCP.

**Figure 10:**
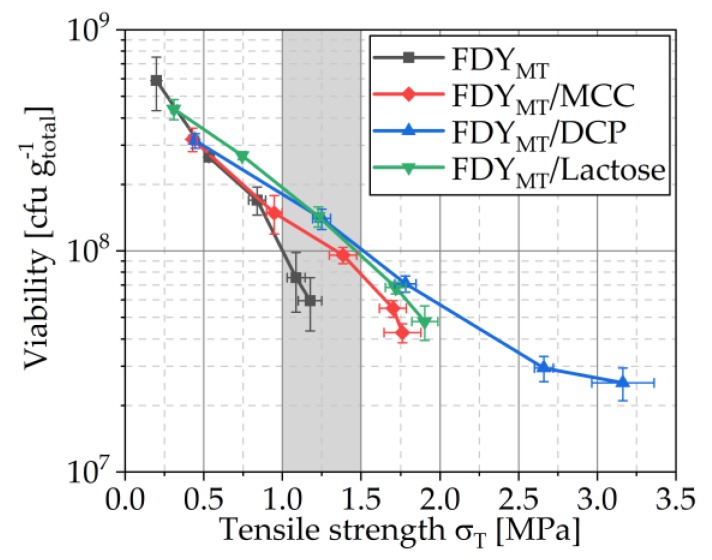
The relationship between viability and tensile strength. The target tensile strength interval (1.0–1.5 MPa) is highlighted. Symbols represent means and error bars standard deviation; *n* = 3.

In tablet production, normally a tensile strength in the range of 1.0 to 1.5 MPa is aimed to ensure sufficient handling stability in combination with an acceptable disintegration time. In Figure 10, the relation of viability of compressed yeast cells (related to total tablet weight) and tensile strength of the tablets is presented. The target tensile strength range is highlighted. This diagram demonstrates how the addition of the excipients could improve the viability of microorganisms during tableting. Despite the lower number of cells in these formulations, the number of viable cells related to the total mass was greater in the target tensile strength interval. This is the result of improving tablet tensile strength by the addition of the excipients, which enables a production of tablets with sufficient tensile strength at lower compaction stresses. Especially for DCP, this effect is so strong that it is overall the most suitable excipient together with lactose.

#### 3.4.6. Friability

The mechanical stress, especially rubbing, during friability testing (rotating of tablets in a drum, European Pharmacopoeia (2.9.7)) caused mass losses of 5.4% and 4.4% for pure FDY_MT_ tablets compacted with 300 and 400 MPa, respectively. The maximal accepted loss of mass according to European Pharmacopoeia (Ph. Eur. 9.3 2.9.7) of 1% could not be attained. However, by adding MCC, DCP and lactose, friability was improved and only 1.0% and 0.9% (FDY_MT_/MCC), 0.5% and 0.4% (FDY_MT_/DCP) and 1.1% and 0.9% (FDY_MT_/Lactose) loss of mass were observed for compaction stresses of 300 and 400 MPa, respectively. To ensure the required abrasion resistance if generally possible, very high compaction stresses above 400 MPa would be necessary for FDY_MT_, highly negatively affecting the survival of the cells as well. By adding MCC, DCP and lactose, lower abrasion was achieved even with lower compaction stress, providing maintenance of viability. Again, the lowest friability for DCP displays its superior property to yield strong tablets due its deformation behavior.

#### 3.4.7. Disintegration

For pure FDY_MT_ tablets, disintegration in purified water took 21 ± 2, 23 ± 1, 25 ± 2, 26 ± 2 and 26 ± 2 min for compaction stresses of approximately 100, 150, 200, 300 and 400 MPa, respectively. This dependency on compaction stress is exceptional; as the porosity decreases, the tensile strength increases drastically with increasing compaction stress. With MCC, DCP and lactose, the disintegration time was 40 ± 3, 40 ± 3 and 25 ± 2 min for compaction stress of approximately 300 MPa and 42 ± 2, 46 ± 4 and 27 ± 1 min for approximately 400 MPa, respectively. Accordingly, compared to pure FDY_MT_ tablets, blending with well soluble lactose did not significantly delayed the disintegration of the tablets, but with MCC and DCP, the disintegration time was longer because of missing solubility and the low or no swelling potential of both excipients [42]. For pharmaceutical application, disintegration of tablets normally should last less than 15 min [14], which was not reached with the tested formulations. Indeed, this is not necessarily a disadvantage because for intestine-targeted tablets a longer disintegration time is preferred to avoid premature release of administered cells [8]. In further formulation development, superdisintegrants or enteric coatings can be studied to directly modify release behavior.

## 4. Conclusions

Industrially relevant process steps for the production of tablets containing viable *S. cerevisiae* cells were investigated regarding the influence of relating process parameters on their survival. Lyophilization was chosen as a mild drying method but strongly affected the yeast cell’s viability during freezing and sublimation. Here, storing the cells at −20 °C until experimental use should be critical, as it could have influenced the survival during the subsequent freezing and sublimation in two contrary directions: (i) Freezing for storage may have pre-stressed the cells, lowering the survival during subsequent freeze-drying or (ii) robust cells were selected by freezing and thawing enhancing survival rates during subsequent lyophilization. Besides the freezing temperature, the use of cryoprotectants was shown as an important parameter influencing cell viability, but further investigations are necessary to improve the understanding of this critical step and to derive process–structure–property relationships.

In part, tableting decreased the viability drastically and survival rates were strongly dependent on applied compaction stress. Indeed, high compaction stresses were necessary to obtain tablets with sufficient tensile strength and friability; but, by addition of direct compression excipients MCC, DCP and lactose, tabletability was improved and higher survival rates for the targeted tensile strength were possible. Here, especially the brittle excipient DCP performed best by presumably forming a coherent network or composite to develop tensile strength while keeping enough space to embed the cells. At the same time, the friability was low when using DCP, but the disintegration was slowed down. Since this is not necessarily detrimental when probiotic microorganisms are administered, DCP can be considered as the most suitable excipient in this study. Whether all microorganisms can benefit from the application of brittle excipients in general should be analyzed in the future.

From the investigated process chain, the compaction during tablet production has turned out to be the most critical step. However, with the type of excipient and the applied stress, two important factors were analyzed that can be used for the improvement of tablet properties, i.e., viability and tensile strength. In addition, the influence of parameters like the compaction speed or mass fraction of the cells should be investigated. Further efforts should be directed towards improving survival for all process steps, but also towards improving the practically equal relevant survival until reaching the site of action, for example by enteric coating of tablets or matrix embedding of cells in swelling polymers to protect them from the harsh conditions in the stomach.

In order to accelerate these improvements, subsequent studies shall focus on providing a detailed understanding of the specific mechanisms of how the cells’ viability is reduced, as well as which cellular structures were harmed depending on the process step and the parameters. It would be desirable to derive a universal process–structure–property relationship from this systematic knowledge, which would enable one to name a suitable manufacturing protocol on the basis of only a few experiments, characterizing the properties of a yeast or bacteria strain that should be formulated, for example, as probiotic drugs.

## Figures and Tables

**Table 1:** Colony forming units and survival rates for freeze-drying *S. cerevisiae* with different cryoprotectant solutions. Rows are sorted in order of decreasing survival rate.

Cryoprotectant $({g\;g}_{\mathrm{CDW}}^{-1}){\;}^{a}$	Viability $\left(10^{9}{\;\mathrm{CFU}\;g}_{\mathrm{CDW}}^{-1}\right)$	Survival Rate (%)	Viability $\left(10^{9}{\;\mathrm{CFU}\;g}_{\mathrm{total}}^{-1}\right)$
40% milk powder	7.50 ± 1.73	42.9	4.66 ± 1.08
25% trehalose + 25% milk powder	7.04 ± 0.09	40.2	4.39 ± 0.06
25% trehalose + 15% milk powder	7.00 ± 0.10	40.0	4.88 ± 0.07
40% trehalose	6.63 ± 0.49	37.9	4.19 ± 0.31
15% dextran	6.33 ± 1.85	36.2	5.70 ± 1.67
25% milk powder	6.15 ± 1.66	35.2	4.81 ± 1.30
15% milk powder	5.78 ± 0.65	33.0	5.23 ± 0.59
25% dextran	5.58 ± 1.59	31.9	4.41 ± 1.29
15% glycerin	5.36 ± 0.16	30.7	4.80 ± 0.14
40% maltose	4.61 ± 0.56	26.3	2.97 ± 0.36
15% trehalose + 15% milk powder	4.50 ± 0.40	25.7	3.21 ± 0.28
40% dextran	4.42 ± 0.13	25.3	2.78 ± 0.09
25% lactose	4.21 ± 0.28	24.1	3.29 ± 0.22
40% lactose	3.65 ± 0.09	20.9	2.27 ± 0.06
25% trehalose	3.50 ± 0.25	20.0	2.81 ± 0.20
15% lactose	3.48 ± 0.41	19.9	3.09 ± 0.36
15% trehalose	3.33 ± 0.13	19.1	3.01 ± 0.12
15% glutamic acid	2.37 ± 0.47	13.5	2.21 ± 0.44
1/5 × PBS	2.37 ± 0.47	13.5	2.44 ± 0.48
Purified water	2.16 ± 0.22	12.4	2.28 ± 0.23
25% glycerin	1.88 ± 0.15	10.7	1.50 ± 0.16
25% maltose	1.50 ± 0.13	8.6	1.21 ± 0.10
15% maltose	1.08 ± 0.10	6.2	1.21 ± 0.11
40% glycerin	0.15 ± 0.01	0.8	1.21 ± 0.07

^a^ except otherwise specified.
